# Genome Sequence of *Helicobacter heilmannii* Sensu Stricto ASB1 Isolated from the Gastric Mucosa of a Kitten with Severe Gastritis

**DOI:** 10.1128/genomeA.00033-12

**Published:** 2013-01-15

**Authors:** Annemieke Smet, Filip Van Nieuwerburgh, Jessica Ledesma, Bram Flahou, Dieter Deforce, Richard Ducatelle, Freddy Haesebrouck

**Affiliations:** a Department of Pathology, Bacteriology and Avian Diseases, Faculty of Veterinary Medicine, Ghent University, Merelbeke, Belgium; b Laboratory of Pharmaceutical Biotechnology, Ghent University, Ghent, Belgium; c NXTGNT Sequencing Facility, Ghent University, Ghent, Belgium; d Next Generation Sequencing Core, The Scripps Research Institute, La Jolla, California, USA

## Abstract

Here we report the genome sequence of *Helicobacter heilmannii* sensu stricto ASB1 isolated from the gastric mucosa of a kitten with severe gastritis. *Helicobacter heilmannii* sensu stricto has also been associated with gastric disease in humans. Availability of this genome sequence will contribute to the identification of genes involved in the pathogen’s virulence and carcinogenic properties.

## GENOME ANNOUNCEMENT

*Helicobacter pylori* is by far the most prevalent *Helicobacter* species in humans with gastric complaints (1). However, long spiral-shaped gastric non-*H. pylori Helicobacter* species (NHPH), also referred to as *Helicobacter heilmannii* sensu lato (2), which naturally colonize the stomachs of animals, have been demonstrated in the gastric mucosa of humans (3). Of the five NHPH shown to be associated with human infections, the genome sequences of *Helicobacter suis* (4), *Helicobacter felis* (5), and *Helicobacter bizzozeronii* (6) are available. Recently, *H. heilmannii* sensu stricto has been successfully isolated from the gastric mucosa of cats (7). This species, highly prevalent in the gastric mucosa of cats and dogs, has been associated with gastritis, gastric and duodenal ulcers, and low-grade mucosa-associated lymphoid tissue lymphoma in humans (2).

Here we report the genome sequence of the *H. heilmannii* sensu stricto type strain ASB1 (LMG 26292) isolated from the gastric mucosa of a kitten with severe gastritis.

The draft genome sequence of *H. heilmannii* sensu stricto ASB1 was performed using 100-base, paired-end reads on the Illumina Hiseq 2000. An Illumina mate-paired library was generated following the Cre-Lox recombination protocol 8. The software tool DeLoxer (8) was used to classify read pairs into 26E+006 true mate-paired reads, 21E+006 paired-end reads, and 25E+006 LoxP-negative reads based on the presence and position of the LoxP sequence in the paired reads. *De novo* assembly was performed using SOAPdenovo, generating 224 scaffolds with a maximum scaffold size of 515,829 bp. These scaffolds were further scaffolded into 5 scaffolds with a maximum scaffold size of 2,136,068 bp using SSPACE (9). Finally a gap-filling step was performed using Gapfiller (10), yielding 1 scaffold of 2,169,401 bp. After reducing long stretches of N’s, the final length of the scaffold was 1,804,623 bp with a 47.4% G+C content. Gene finding and automatic annotation were done using the RAST server (11).

The *Helicobacter heilmannii* sensu stricto ASB1 genome contains 1,918 protein-coding sequences (CDSs) with an average length of 933 bp, 41 tRNA genes, and 9 rRNA genes. A putative function could be predicted for 1,183 (62%) of CDSs, whereas 735 (38%) of CDSs were annotated as hypothetical proteins.

Comparison with completed genome sequences of *H. pylori* showed that the *H. heilmannii* sensu stricto genome contains several genes encoding homologues of known *H. pylori* virulence factors, such as the gamma-glutamyl transpeptidase GGT, the immunomodulator NapA, the flavodoxin FldA, the plasminogen-binding proteins PgbA and PgbB, the collagenase PrtC, the carcinogenic factor Tipα, the proline oxidase PutA, and the secreted serine protease HtrA. This genome encodes several outer membrane proteins but lacks the Bab and Sab adhesins which are important in *H. pylori*. It possesses a complete comB system conferring natural competence but lacks a Cag pathogenicity island as well as homologue genes encoding a vacuolating cytotoxin VacA.

The availability of the *H. heilmannii* sensu stricto genome sequence will provide a platform to identify new genes that may contribute to this pathogen’s virulence and carcinogenic properties as well as for further analysis of genomic variability, plasticity and bacterial evolution.

### Nucleotide sequence accession number. 

The genome sequence of the *Helicobacter heilmannii* sensu stricto type strain ASB1 (LMG 26292) has been deposited in the EMBL database under the accession number HE984298 (project identification number PRJEB367).
